# Global assessment of swallow function (GASF) following VMAT radiotherapy for head and neck squamous cell carcinoma

**DOI:** 10.1016/j.tipsro.2024.100272

**Published:** 2024-08-30

**Authors:** Kate Toft, Kirsty McLachlan, Mark Winton, Karen Mactier, Nadine Hare, Claire Nugent, Lucie Wincott, Devraj Srinivasan, Joanna Mackenzie, Bill Nailon, David Noble

**Affiliations:** a Edinburgh Cancer Centre, NHS Lothian, Western General Hospital, Crewe Road South, Edinburgh EH4 2XU, UK; b Department of Speech and Hearing Science, Queen Margaret University, Queen Margaret University Drive, Musselburgh EH21 6UU, UK; c School of Cancer Sciences, College of Medicine, Veterinary Medicine and Life Sciences, University of Glasgow, University Avenue, Glasgow G12 8QQ, UK; d The Institute for Imaging, Data and Communications (IDCOM), School of Engineering, The University of Edinburgh, Edinburgh EH9 3BF, UK; e The Department of Biomedical Engineering, Fulton Building, University of Dundee, Dundee DD1 4HN, UK; f Edinburgh Cancer Research Centre, The Institute of Genetics and Cancer, Crewe Road South, University of Edinburgh, Edinburgh EH4 2XU, UK

**Keywords:** Dysphagia, Head and neck cancer, Outcomes, Radiotherapy

## Abstract

**Aim:**

This study aimed to conduct a global assessment of swallow function (GASF) using a range of swallow outcome tools, in a cohort of patients pre- and post-treatment with image-guided volumetric modulated arc therapy (VMAT) radiotherapy.

**Materials and methods:**

All patients receiving radical (chemo)radiation for SCC of the larynx, oro-, hypo- or nasopharynx between October 2016 – 2021 were eligible for inclusion.Patients were treated with VMAT radiotherapy according to institutional and national protocols.

Patients underwent GASF pre- and 6 months post-treatment. Data were collated from the MD Anderson Dysphagia Inventory (MDADI), the Functional Oral Intake Scale (FOIS), the Performance Status Scale-Head and Neck: Normalcy of Diet scale (PSS-NoD), the 100 ml water swallow test (WST) for capacity and maximal interincisal opening (MIO).

**Results:**

One hundred and seventy-five patients were included. 55.2 % of patients experienced a fall in MDADI-Composite score greater than the published meaningful clinical important difference (MCID).A trend for a decrease in FOIS score reflects a decrease in range of diet textures and increase in reliance on enteral feeding at 6 months post-treatment.Mean PSS-NoD score decreased reflecting increased restriction in diet textures.20 patients’ WST capacity improved by the minimal clinically important difference of > 4mls whilst 37 % of patients experienced a decrease in WST capacity of 4mls or more. 12.6 % of patients developed trismus following radiotherapy.

**Conclusions:**

This paper adds new detail to the understanding of the decline in measured eating, drinking and swallowing function that patients treated with VMAT radiotherapyexperience at 6 months post treatment. However, gaps are highlighted in the evidence base in terms of interpretation of swallow outcomes tool scores; future research in HNC should include ongoing discussion and development around robust outcomes tools and data collection.

## Highlights

• VMAT-treated HNSCC patients tend to experience a decline in measured eating, drinking and swallowing function at 6 months post treatment.
• Eating, drinking and swallowing was impacted across a wider range of metrics than previously reported: swallow-related quality of life, range of oral diet textures, reliance on enteral feeding, jaw opening, and swallow capacity.
• 55.2 % patients experienced a fall in MDADI-C of at least 10 points.
• 12.6 % of patients developed trismus following radiotherapy.

## Introduction

Head and neck cancer (HNC) is the 8th most common malignancy in the UK, and 90 % of these cancers are squamous cell carcinomas (HNSCC) [1]. These tumours occur in a wide range of sites within the upper aerodigestive tract. Swallowing dysfunction, or dysphagia, is highly prevalent in this population due to tumour burden and/or treatment toxicity [2]. Patients are keen to minimise this because of its implications for function and quality of life [3], [4]. Several clinical trials have focussed on de-escalation to reduce functional side-effects and preserve survival outcomes [5], [6], [7], [8], [9]. These trials rely on capturing swallow outcome data using a variety of assessment tools to illustrate change from the pre-treatment to post-treatment survivorship phase. Current guidelines stipulate that swallow outcome measurements [10], [11] include multiple measures capturing aspects such as physiological impairment, mealtime function and dysphagia related quality of life.

Commonly used measures include instrumental swallow assessments, clinician-rated scales of oral intake such as the functional oral intake scale (FOIS) [12] and the performance status scale (PSS)-Head and Neck [13], and patient reported outcomes tools such as the MD Anderson Dysphagia Inventory (MDADI) [14] which measures dysphagia-related quality of life.

Several studies highlight the impact of oncological treatment on swallow function; however these data include a heterogeneous population, mixed treatment modalities (i.e. surgical and oncological), different cancer sites/stages, large variation in post-treatment data capture and variability of outcomes [9], [15]. Furthermore, studies are often retrospective in nature and many report swallow outcomes from cohorts treated with outdated radiotherapy (RT) techniques [16], [17], [18].

The aim of this study was to conduct a global, multi-tool assessment of swallow function (GASF) before and after treatment in a large cohort of patients with HNSCC all treated with VMAT radiotherapy with radical intent.

## Methods

### Ethical considerations

The study is a retrospective review of data collected during routine clinical practice, registered as a service evaluation project with the host institution. Information governance procedures were approved as part of the local Cancer Information Programme (project number CIR22065).

### Participants and recruitment

Prospective, consecutive swallow outcome data was collected for all patients with HNSCC of the naso-, oro-, hypo-pharynx or larynx receiving VMAT RT with or without concomitant chemotherapy (cRT) with curative intent, treated between 2016 and 2021, at two timepoints: pre-treatment and 6 months post-treatment. Other authors have suggested that the 6 month timepoint correlates well with longer term outcomes [19], and this timepoint also coincides with standard clinical follow up protocols, allowing patient concordance with assessments and low attrition rates. All patients received prophylactic swallowing exercises, which they commenced prior to their treatment, and were supported to continue on-treatment.

Patients with biopsy proven HNSCC in a neck lymph node who, after extensive investigation, had no demonstrable primary site of disease (T0), and those who had undergone diagnostic surgical procedures (e.g. diagnostic tonsillectomy) prior to definitive RT/cRT were included, but patients who had undergone any radical surgical procedure (e.g. neck dissection) were excluded. A summary of inclusion and exclusion criteria are presented in Table 1.

**Table 1:** Inclusion and exclusion criteria for the participants to the study.

**Inclusion criteria**	**Exclusion criteria**
Age ≥ 18 years	Primary tumour of the oral cavity
Primary tumour of the nasopharynx, oropharynx, larynx or hypopharynx, or true unknown primary HNSCC	Definitive surgery with adjuvant oncological treatment
Primary (chemo)radiotherapy treatment with curative intent	Patients with known residual or recurrent disease at the 6 m post-treatment timepoint

HNC MDT Speech & Language Therapists (SLTs) collected outcomes data during routine clinical consultations.

### Treatment details

All patients treated for HNSCC of the oropharynx, hypopharynx and larynx received 65 Gy in 30 fractions, whilst patients with nasopharyngeal disease received 70 Gy in 33 fractions. Contouring and RT planning was conducted according to international guidelines [20], [21], [22], recent clinical trial protocols [8], [23], [24] and local clinical guidelines. Patients undergoing concomitant chemotherapy received either up to 2 cycles of cisplatin 100 mg/m^2^, or up to 2 cycles of carboplatin AUC5 in weeks 1 and 5 of radiotherapy treatment. Patients receiving radical chemoradiation were recommended to have a prophylactic Radiologically Inserted Gastrostomy (RIG) tube inserted. Patients receiving radical radiotherapy only with hidose volume or risk factors such as baseline dysphagia were offered prophylactic RIG; all other patients would receive reactive nasogastric feeding if necessary.

### Outcomes tools

Data from five swallow outcome tools wereassessed: the MDADI, the FOIS, the PSS-Head and Neck: Normalcy of Diet scale (PSS-HN NoD), the 100 ml water swallow test (WST) and maximal interincisal opening (MIO).

#### MDADI

The MDADI [14] is a patient reported outcome measure of dysphagia related quality of life developed specifically for use with patients with HNC. The MDADI gives two scores, a global score (MDADI-G) and a composite score (MDADI-C), where the maximum score is 100, reflecting better dysphagia-related quality of life, and the minimum score is 20.

#### FOIS

The FOIS [12] is a clinician-rated measure which quantifies degree of oral intake versus enteral feeding. Scores range from 1 to 7 with 7 representing ‘normal’ function and 1 indicating a total tube dependence with no oral intake.

#### PSS-HN- normalcy of diet

The PSS H&N NoD is a clinician-rated scale reflecting patients’ ability to eat a range of food textures. Scores range from 0 to 100 [13] with 100 representing no restriction in oral diet.

#### WST

The WST is a timed swallowing test which involves a patient swallowing 100 ml of water in the fastest time possible. A swallow ‘capacity’ metric calculated from this test (i.e. mls swallowed per second) is presented in this paper [25], [26].

#### MIO

MIO is a measure of jaw opening in millimetres. SLTs used a Therabite jaw measurer for this purpose. The accepted cut off for reduced jaw opening (trismus) in the literature is ≤ 35 mm [27].

### Analysis

Baseline demographic, disease, and treatment data were prospectively collected and stored within a password protected Microsoft Excel^TM^ spreadsheet on a secure institutional server. Data analyses were completed using R version 4.3.1 [28]. Data wrangling was performed using the Tidyverse collection of packages version 2.0.0, Glue version 1.7.0 and janitor 2.2.0 packages. Descriptive statistics were obtained using the skimr package 2.1.5. Differences between baseline and 6-month mean scores were assessed using the Wilcoxon Sign Ranked test, with p-values < 0.05 taken to infer statistical significance.

## Results

### Baseline clinical characteristics

The sample consisted of 134 males and 42 females with HNSCC, with a mean age of 60.7 years (range 36–87 years). Tumour and node classification and location of primary site are detailed in Table 2. Twelve patients received neo-adjuvant chemotherapy.

**Table 2:** Patient characteristics.

**Characteristics**	**Number**	**%**
*Sex*		
Male	134	76.1
Female	42	23.9
*Age*		
18–65	122	69.3
> 65	54	30.7
*Tumour Classification (AJCC 7th Edition)*		
T0	8	4.6
T1	44	25
T2	65	36.9
T3	32	18.2
T4	27	15.3
*Node Classification(AJCC 7th Edition)*		
N0	53	30.1
N1	39	22.2
N2	80	45.5
N3	4	2.3
*Primary Site*		
Oropharynx	118	67.1
Hypopharynx	9	5.1
Larynx	37	21.0
Nasopharynx	4	2.3
Unknown Primary	8	4.6
*Treatment Modalities*		
Radiotherapy Only	62	35.2
Concurrent ChemoRadiotherapy	114	64.8

### Swallow outcomes data

Not every patient completed every pre-treatment 6-month assessment. The number of datapoints per outcome measure, and the number of paired assessments (pre- and post-treatment) are presented in Table 3. Median scores were significantly lower 6 months post-treatment in all measured outcomes.

**Table 3:** Swallow outcomes data summary.

**Outcome measure**	**Baseline**				**6 months**				**p value**
	**n**	**range**	**Mean (95 % CI)**	**Median (IQR)**	**n**	**range**	**Mean (95 % CI)**	**Median (IQR)**	
**MDADI – G**	168	20–100	88.7 (85.8–91.6)	100 (80–100)	161	20–100	75.5 (71.5–79.5)	80 (40–100)	< 0.001
**MDADI – C**	167	22–100	87.2 (84.9–89.5)	94.7 (78.9–100)	151	28.4–100	73.2 (70.3–76)	73.7 (60–87.8)	< 0.001
**PSS-HN NoD**	175	20–100	88 (84.9–91.1)	100 (90–100)	174	0–100	75.3 (71.8–78.8)	90 (50–90)	< 0.001
**FOIS**	174	2–7	6.69 (6.59–6.8)	7 (7–7)	173	1–7	6.14 (5.96–6.32)	7 (6–7)	< 0.001
**MIO**	159	18–67	45.7 (44.4–47)	46 (40–51.8)	137	7–65	41.7 (40.3–43.1)	42 (35.8–46.2)	< 0.001
**WST Capacity (mls/sec)**	168	1–50	15.4 (14.1–16.7)	14.4 (8.75–20.2)	143	0.1–50	13.1 (11.7–14.4)	12.5 (6.7–16.7)	< 0.001

### Swallow outcomes data

#### MDADI

**Fig. 1:**
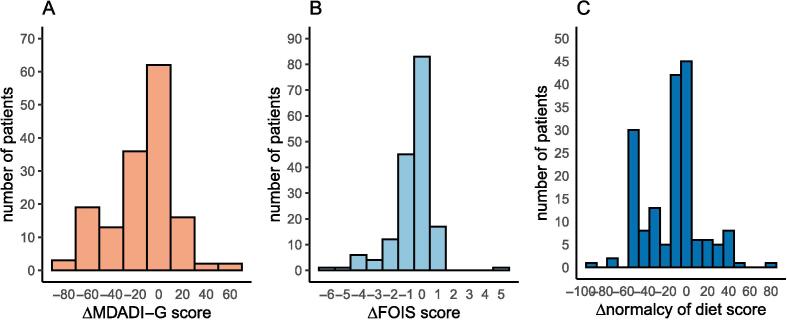
Swallow outcome score changes.

Mean baseline scores were 88.7 (95 % CI 85.8–91.6) and 87.2 (95 % CI 84.9–89.5) for MDADI-G and MDADI-C respectively. At 6 months post-treatment, mean MDADI-G score had fallen to 75.5 (95 % CI 71.5–79.5): a decrease of 13.2, and mean MDADI-C to 73.2 (95 % CI 70.3–76) for: a decrease of 13.5. Both changes were statistically significant. Fig. 1A illustrates the MDADI-G score changes between baseline and 6 months across the cohort.

**Fig. 2:**
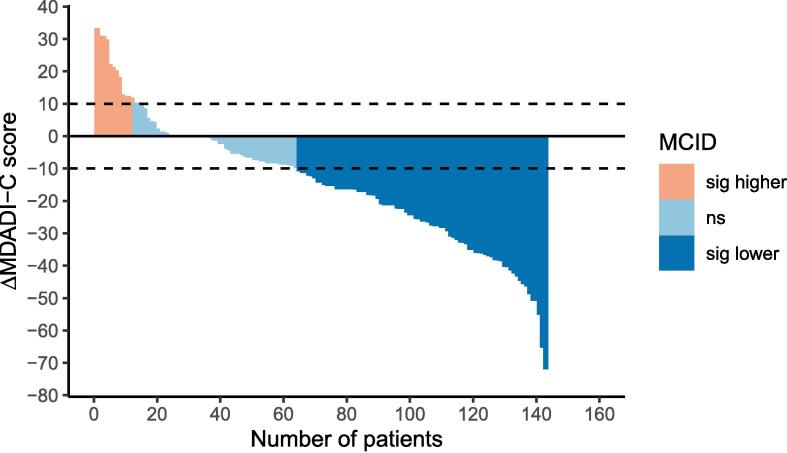
MDADI-C score change.

The negative trend in MDADI-C, i.e. a decrease in dysphagia-associated quality of life at 6-months post-treatment, is demonstrated in the waterfall plot in Fig. 2. A minimal clinically important difference (MCID) of 10 points has been suggested for MDADI-C. Applying this definition to this cohort, 8.4 % of patients had a clinically important improvement in MDADI-C score whilst 55.2 % experienced a clinically important fall in MDADI-C.

#### FOIS

At baseline the mean FOIS score was 6.69 (95 % CI 6.59–6.8) and at 6 months post-treatment the mean score was 6.14 (95 % CI 5.96–6.32), a fall of 0.55. Fig. 1B shows the distribution of FOIS score changes in patients who had both baseline and 6-month data for analysis. The difference between baseline and 6 months FOIS scores was also statistically significant, which reflects a decrease in range of diet textures and increase in reliance on enteral feeding at 6 months post-treatment.

#### PSS – HN normalcy of diet

Mean PSS-NoD score at baseline was 88 (95 % CI 84.9–91.1) with a decrease to a mean of 75.3 (95 % CI 71.8–78.8), a fall of 12.7. This difference was also statistically significant. A histogram of change in PSS-NoD is shown in Fig. 1C. The majority of patients experienced a decline in swallowing function based on this score and 34/174 (24.3 %) saw a fall of 50 points or more, reflecting a marked restriction in diet textures post-treatment.

#### MIO

**Fig. 3:**
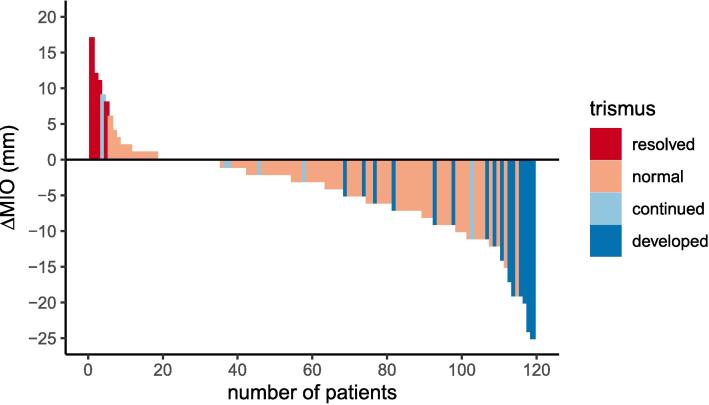
MIO change.

MIO measurements at baseline ranged from 18-67 mm with a mean of 45.7 mm (95 % CI 44.4–47). At 6 months post-treatment the mean measurement was 41.7 mm (95 % CI 40.3–43.1), with a range of 7–65 mm. At baseline, 24/159 patients (15.1 %) recorded an MIO of 35 mm or less, which is regarded as clinical trismus [27]. At 6 months post-treatment, this had increased to 34/137 (24.8 %). Change in MIO for the 119 patients with complete data is shown in Fig. 3.

Ninety-two (77.3 %) patients did not have trismus based on MIO either at baseline or at 6 months, and for 4 (3.4 %), trismus resolved after treatment. However, 8 (6.7 %) continued to experience trismus, and 15 (12.6 %) had developed trismus by 6 months post-treatment.

#### Water swallow test

**Fig. 4:**
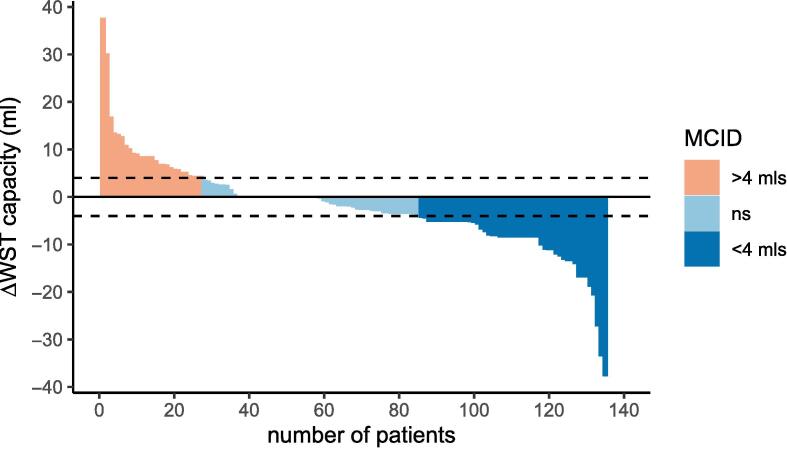
Waterfall plot of WST Capacity change.

At baseline the mean WST capacity score was 15.4 ml/sec (95 %CI 14.1–16.7). At 6 months post-treatment the mean score had fallen to 13.1 (95 % CI 11.7–14.4), the difference between means showing a statistically significant decrease in swallow capacity of 2.3mls per swallow on average. WST differences for 135 patients with paired data are shown in Fig. 4.

This waterfall plot shows that 27 (20 %) improved WST capacity by the MCID of > 4mls, whilst 50 (37 %) experienced a decrease in WST capacity of 4mls or more.

## Discussion

This study is the first to present global, multi-tool assessment data of swallow function before and after treatment in a large cohort of patients with HNSCC treated with VMAT radical radiotherapy. A wide range of swallowing data from several outcome measures gives a more global and nuanced understanding of the impact of HNSCC treatment on patients’ swallowing function and their experience of eating and drinking.

### Summary of key findings

We describe changes in scores across all swallow outcomes tools between baseline and 6 months post-treatment with wide variability in score ranges. All scores were significantly worse at 6 months post-treatment. The large interpatient variation in scores, in keeping with other studies [9], [16], [29] reflecting the challenges of measuring swallow outcomes in the HNC population.

#### MDADI

As with previous published data [9], [16], MDADI-G and −C scores in this patient cohort were lower at 6 months post-treatment, reiterating the detrimental impact on swallowing-related quality of life. There was a substantial decrease between mean MDADI-C scores in this cohort, more than the published MCID for this tool [30].

Nutting *et al.*[9] presented their MDADI data with reference to the published MCID (i.e. the proportion of patients who had a ≥ 10 change). The proportion of patients in our cohort whose outcomes improved or declined by according to the published MCID is similar to those seen in DARS [9]. Further, the mean baseline MDADI-C score in our cohort (87.2) is similar to those in DARS (87.6 in the S-IMRT group, 87.2 in the DO-IMRT group).

Table 4 below compares our GASF MDADI-C data with the DARS study [9].

**Table 4:** Comparison of MDADI-C score change with respect to MCID.

		Better			Worse		
		DO-IMRT N (%)	S-IMRT N (%)	Chi-squared p value	DO-IMRT N (%)	S-IMRT N (%)	Chi-squared p value
Nutting 2023 data	MDADI-C at 6 *m*	6 (10.7)	8 (14.5)	0.54	34 (60.7)	32 (58.2)	0.79
GASF data	MDADI-C at 6 *m*	15 (10.49)			79 (55.24)		

#### FOIS

There was an overall trend for downward change in FOIS scores, and this is mirrored in other work published by Charters *et al.* [31]. FOIS is routinely used in HNC practice [32], however predicting a clinically significant change is challenging as the scores on the scale do not represent interval data. For example, the difference between a score of 1 and 2 (‘No oral intake’ vs ‘Tube dependent with minimal/inconsistent oral intake‘) may not have the same impact for patients as a difference between a score of 6 and 7 (‘Total oral intake with no special preparation but must avoid specific foods or liquid items’ vs ‘Total oral intake with no restrictions’). In terms of clinical utility the FOIS has the advantages of being quick to administer and score, and providing information on reliance of enteral feeding and nil by mouth (NBM) status.

#### PSS-HN NoD

Mean PSS NoD score decreased by 12.7 between timepoints. There is limited comparison with other studies due to differences in number of patients reported, timepoints and methods of analysis. this study shows a greater deterioration than the change reported elsewhere (n = 114) [16] where mean score fell by 8 points.

An MCID for the PSS NoD has yet to be published so it is difficult to put this into functional context in terms of clinical impact. The PSS consists of ordinal categories; and similar to the FOIS a difference for example in 10 points cannot be assumed to have equal functional impact across the entirety of the scale There is also the possibility that dietary restrictions reflected in the PSS NoD may result from other factors such as reduced dentition or xerostomia [26] rather than dysphagia per se. The PSS is however quick to administer and score, gives an indication of NBM status and is widely used and recognised in the HNC clinical and research community.

#### MIO

The mean pre-treatment MIO for our cohort was 45.7 mm, similar to Aghajanzadeh *et al.* who reported a pre-treatment mean MIO of 51.5 mm in 211 HNC patients treated with radiotherapy in a more mixed patient group [33]. It should be noted that baseline MIO measurements may be impacted by pre-treatment procedures such as biopsies and dental extractions.

In the GASF cohort 6 m post treatment mean MIO fell to 41.7 mm (8.8 % change) compared with 41.4 mm (19.6 % change) in the Aghajanzadeh cohort. Thirty-four (24.8 %) GASF patients presented with trismus at the 6 m point, comparable with data published by van der Geer *et al.* [34] who reported a 23.6 % post-treatment trismus prevalence of 23.6 %. In our cohort 12.6 % of patients developed trismus following treatment; previously published papers however do not present paired pre/post treatment data for comparison.

#### WST

In the GASF cohort, the difference between baseline and 6-month means was 2.3mls per second: less than the published MCID [26]. The wide data range and IQR reported at both timepoints highlights a spread in our cohort, which makes it difficult to link functional meaning to these changes.

The 100 ml WST has been validated on a HNC patient population and has been found to have good test–retest reliability [35], is fast to administer and requires no specialist equipment. However, there is a lack of clarity in the evidence base about the clinical interpretation of the scores. Scores have been shown to vary by age and gender [35]. Also given the nature of the test, not all HNC patients are able to comply depending on their functional swallow status and aspiration risk [36]. Nonetheless, the statistically significant fall in WST capacity observed in patients between baseline and 6 months suggests it has some utility in quantifying biomechanical changes in swallowing function following treatment.

### Strengths and limitations of study

#### Strengths

The dataset is considerably larger than a number of other published series that have assessed prospectively collated swallow function data following radical radiotherapy for HNC [9], [16], [31]. Furthermore, in contrast to many this study reports outcomes from a cohort of patients treated with contemporary image guided VMAT. In addition, this study is unique by providing a global assessment of swallow function and its appraisal of the strengths, weaknesses and clinical implications of the different outcome scores presented. We anticipate that the study will contribute to the ongoing discussion in head and neck practice around the practicalities and importance of outcomes data collection and their use to improve patient care.

#### Limitations

This study presents only a single follow up timepoint (6 months), which does not give a longitudinal representation of how swallowing function changes over longer term follow up. Whilst some authors have suggested that function at 6 months generally reflects longer term outcomes [19] − and this understanding concurs with our experience in the clinic − recent results from the DARS study contradict this notion, although interestingly this seemed partially to depend upon radiotherapy treatment technique [9]. In the standard IMRT technique cohort – the treatment technique also used for patients in this study –there was little change in swallowing function between 6 and 12 months, whilst those patients who were treated with dysphagia-optimised IMRT saw greater improvements between 6-month function and longer term outcome. Nonetheless we recognise that this study lacks truly long term follow up data.

We note also that instrumental swallow assessment data was not included in the dataset, as this would only have been available for a small proportion of patients, reflecting the fact that our data was collected as part of routine SLT clinical practice. In addition, we recognise that other RT-induced side-effects such as xerostomia can also negatively impact swallowing function, and this data has not been routinely collected in our cohort. However, we suggest that the GASF approach described in this study partially mitigates this limitation, as the detailed granular data collected helps clinicians to disentangle the different contributing components to swallowing dysfunction at the level of the individual patient.

### Clinical implications of results and conclusions

This paper adds new detail to the current understanding of the impact of VMAT radiotherapy on swallow function outcomes for people with HNC. Swallow outcome data can be used clinically to flag unmet need, guide and evaluate therapeutic swallow intervention, and inform patient counselling and consent. All the tools discussed in this paper are routinely used in HNC clinical and research practice; however, there are gaps in the evidence base in terms of facilitating interpretation of scores. These gaps include a lack of a standardised ‘core outcome set’, consensus on ideal post-treatment outcomes datapoints, and limited knowledge of tools MCIDs, psychometric properties and clinical utility. This results in reduced potential for swallowing outcome data to inform meaningful discussions with patients and the wider MDT about functional outcomes and quality of life following HNC treatment.

An additional concern about existing outcome measures is the potential for them to exclude patients whose dysphagia is so severe that they are completely feeding tube dependent (e.g. MDADI and WST). A significant cohort of HNC patients therefore will be underrepresented in outcomes studies as they are unable to complete the full suite of measures.

This study shows that collecting comprehensive outcomes data is achievable within routine clinical practice. Further work and ongoing discussion within the HNC community is required to develop more robust outcomes tools for universal data collection. Through wider collection of HNC data there will be the opportunity to power more detailed analyses and produce information that will have a positive impact on patient care.

## Suggested reviewers

Professor Jo Patterson, Professor of Speech & Language Therapy, School of Health Sciences, Institute of Population Health, University of Liverpool Joanne.Patterson@liverpool.ac.uk.

Prof Sara Faithful, Professor of Cancer Nursing Practice, University of Surrey, sara.faithfull.prof@gmail.com.

Prof Helen McNair, lead research radiographer at The Royal Marsden NHS Foundation Trust and Reader in Translational Therapeutic Radiography at The Institute of Cancer Research, London. Helen.McNair@rmh.nhs.uk.

## CRediT authorship contribution statement

**Kate Toft:** Conceptualization, Investigation, Resources, Data curation, Methodology. **Kirsty McLachlan:** Conceptualization, Investigation, Resources, Data curation, Methodology. **Mark Winton:** Conceptualization, Data curation, Methodology. **Karen Mactier:** Writing – review & editing, Visualization. **Nadine Hare:** Investigation, Resources, Data curation. **Claire Nugent:** Investigation, Resources, Data curation. **Lucie Wincott:** Investigation, Resources, Data curation. **Devraj Srinivasan:** Resources, Writing – review & editing. **Joanna Mackenzie:** Resources, Writing – review & editing. **Bill Nailon:** Conceptualization, Writing – review & editing. **David Noble:** Conceptualization, Resources, Formal analysis, Data curation, Methodology.

## Declaration of competing interest

The authors declare that they have no known competing financial interests or personal relationships that could have appeared to influence the work reported in this paper.
